# Ecological dichotomies of solar energy expansion: resilience in arid regions versus fragility in humid ecosystems

**DOI:** 10.3389/fpls.2025.1549519

**Published:** 2025-03-04

**Authors:** Jianhua Xiao, Panxing He, Yong Li, Mingjie Shi, Yang Li, Jun Ma

**Affiliations:** ^1^ Key Laboratory of Ecological Safety and Sustainable Development in Arid Lands, Northwest Institute of Eco-Environment and Resources, Chinese Academy of Sciences, Lanzhou, China; ^2^ Ministry of Education Key Laboratory for Biodiversity Science and Ecological Engineering, School of Life Sciences, Fudan University, Shanghai, China; ^3^ Jurong Power Generation Branch, Huaneng Jiangsu Energy Co., Ltd, Zhenjiang, China; ^4^ Xinjiang Key Laboratory of Soil and Plant Ecological Processes, Xinjiang Agricultural University, Urumqi, China; ^5^ College of Agriculture, Yanbian University, Yanji, China

**Keywords:** utility-scale solar energy, ecological dichotomies, vegetation greenness, scale effect, arid regions

## Abstract

The deployment of Utility-Scale Solar Energy (USSE) systems is increasingly recognized as a cornerstone strategy in mitigating climate change. However, the environmental ramifications of such extensive developments remain the subject of considerable debate, with marked regional variability in their ecological effects, particularly across different biomes. As such, there is a pressing need for comprehensive, systems-level investigations to evaluate the multifaceted environmental impacts of USSE in both arid and humid ecosystems. Here, we undertake an exhaustive assessment utilizing a high-resolution (10 m) dataset of photovoltaic (PV) station distributions across China, complemented by Landsat-derived NDVI remote sensing data from 2019 to 2023. This approach facilitates the quantification of the dynamic effects of PV infrastructure development on vegetation greenness (NDVI_mean_ and NDVI_max_), and allows for the assessment of scale-dependent ecological responses across two contrasting regions: the arid zone of Ningxia and the humid zone of Anhui. Our results indicate that in the arid region, the construction of PV facilities has a negligible effect on vegetation greenness, with inter-annual variations in NDVI_mean_ remaining consistently below 0.05, and no discernible change in NDVI_max_. In contrast, PV development in the humid region led to a dramatic deterioration in vegetation greenness, with NDVI_mean_ declining sharply from 0.42 to below 0.20—representing a reduction of over 50%, particularly during the growing season (April to October). Furthermore, the relationship between the scale of PV installations and their ecological impact in the humid region was characterized by a pronounced non-linearity, with large-scale PV plants (spanning >10,000 pixels) causing near-total vegetation collapse, driving NDVI toward near-zero. Collectively, these findings suggest that the sparse vegetation and enhanced microclimatic regulation characteristic of arid ecosystems provide greater resilience to external disturbances, whereas the high-biomass vegetation typical of humid regions is significantly more vulnerable to perturbations. Based on these insights, we advocate for the strategic prioritization of arid regions with greater ecological adaptability for future USSE development, alongside the incorporation of ecological restoration measures and the optimization of facility scale to mitigate potential environmental disturbances. Our study emphasizes the need for a synergistic approach to optimize both energy transition and ecological conservation in the context of regional variability, offering a solid scientific basis for the national-scale planning and site selection of photovoltaic energy projects.

## 1 Introduction

In recent years, China has vigorously advanced the large-scale deployment of photovoltaic (PV) systems, particularly in desert, gobi, and desertification regions, as part of its overarching strategy to achieve carbon neutrality and drive the energy transition (Wang et al., 2023, 2024b). By 2023, China’s cumulative solar power capacity has attained a global leadership position, accounting for more than 45% of the world’s total PV installations (IEA, 2023). There is no doubt that the expansion of Utility-Scale Solar Energy (USSE) systems has made a substantial contribution to diminishing reliance on fossil fuels and addressing the global climate crisis (De Marco et al., 2014; Hernandez et al., 2014). However, the rapid proliferation of such systems has been accompanied by a host of potential ecological disturbances, encompassing land-use conflicts and localized alterations in microclimates, which may have far-reaching and multifarious consequences (Armstrong et al., 2016; Zhang et al., 2023).

The environmental drawbacks of USSE development are particularly pronounced. For instance, the construction of USSE facilities often necessitates extensive clearing of surface vegetation, which can lead to soil erosion, reduced precipitation infiltration, and disruptions in local hydrological systems (Sánchez-Zapata et al., 2016; Zhang et al., 2023). In desert regions, the installation of PV systems not only results in a reduction of plant populations but also significantly alters soil structure (Choi et al., 2020), thereby diminishing the region’s carbon storage capacity. Furthermore, the placement of PV panels over agricultural land can obstruct crops’ access to sunlight, thereby limiting photosynthesis and ultimately reducing agricultural yields (Weselek et al., 2019). For example, crops such as cucumbers, which require high light intensity, exhibit significant yield reductions under the shading effect of PV panels (Marrou et al., 2013). More critically, the expansion of PV installations in humid regions may exacerbate land-use conflicts, directly competing with objectives of food production and forest conservation, leading to long-term instability in land-use patterns (Song et al., 2024).

In contrast, several studies have underscored the potential positive environmental impacts of USSE systems (Turney and Fthenakis, 2011; Uldrijan et al., 2021). The shading effect of PV panels can reduce soil surface temperature and mitigate water evaporation, thereby enhancing plant water-use efficiency and increasing drought resilience (Yue et al., 2021). This effect could positively influence crop yields in drought years, with drought-tolerant crops such as maize exhibiting increased productivity under shaded conditions (Amaducci et al., 2018). Furthermore, the establishment of PV infrastructure on degraded lands may foster ecological restoration, particularly when integrated with restoration techniques like vegetation regeneration (Wang and Gao, 2023), potentially facilitating the recovery of carbon sequestration capacity (Li et al., 2024). It is evident that extant research largely neglects the variation in regional ecological characteristics when examining the environmental impacts of PV installations. This oversight is especially pronounced in the context of contrasting ecosystems, such as arid and humid regions, where a dearth of comparative studies has led to significant disparities in the scholarly discourse regarding the ecological benefits of USSE deployment. In light of intensifying climate change, environmental pressures in different ecological regions exhibit pronounced divergence. Arid regions are particularly vulnerable to external perturbations due to reduced precipitation and water scarcity (Huang et al., 2017). In contrast, humid regions are more susceptible to the repercussions of land-use changes, as they are marked by increased precipitation variability and the fragility of high-biomass vegetation (Allan and Soden, 2008). These regional discrepancies present a novel dimension for exploring the complex ecological responses to USSE development. These regional discrepancies underscore the importance of exploring the ecological responses to USSE development in different ecosystems. This is particularly relevant for arid and humid regions, where climate change exacerbates environmental pressures and increases regional divergence.

Traditionally, the environmental impact assessments of PV installations have predominantly relied on ground-based observations and localized experiments (Yue et al., 2021; Hurduc et al., 2024). While these methods provide direct data support, their spatial coverage is inherently limited, making it challenging to comprehensively monitor the ecological effects of large-scale PV installations. With the advancement of remote sensing (RS) technology, satellite-based monitoring approaches have gradually become the predominant methodology for studying the environmental impacts of PV installations (Zhang et al., 2021; Wang et al., 2024a). Recently, the PV facility benchmark database developed by Feng et al. (2024) has emerged as a crucial resource for RS studies. This database systematically integrates multi-source RS dataset, offering high-resolution distribution information on PV facilities across China at a national scale.

Vegetation greenness indices have become a widely accepted tool for assessing the impact of PV installations on vegetation cover (Xia et al., 2023). For instance, Xia et al. (2024) integrated high-resolution RS imagery with Geographic Information System analysis to investigate the reconfiguration of ecological services on degraded lands following PV development. Their study suggests that, under proper management, the deployment of USSE systems in arid and semi-arid regions can significantly promote land revegetation and enhance carbon sequestration capacity (Xia et al., 2024). Additionally, RS products have been utilized to monitor the dynamic changes in vegetation condition surrounding PV facilities, revealing a subtle yet discernible increase in vegetation coverage during the growing season post-installation (Wang et al., 2024b). Despite a few recent studies employing RS technologies to capture the disturbance processes associated with PV facilities, there remains a conspicuous research gap in using such techniques to explore the ecological responses to USSE deployment across varying regional contexts.

The ecological impacts of PV installations exhibit significant regional variability, largely shaped by local water-thermal conditions (Hurduc et al., 2024; Li et al., 2024), with the scale of PV plant construction potentially inducing non-linear effects on ecosystem health. Based on this, we hypothesize that the environmental impact of PV installations is contingent upon the ecological characteristics of the region in which they are situated. In arid regions, low vegetation cover and high environmental adaptability prevail. As a result, the negative effects of PV installations may be relatively modest. Under certain conditions, they could even facilitate ecological restoration. In contrast, in humid regions, where high biomass and complex ecological services are characteristic, the development of PV infrastructure is likely to incur a greater ecological cost. Furthermore, we posit that variations in the scale of PV installations could either exacerbate or mitigate these disparities, with large-scale projects potentially intensifying land disturbances, while smaller-scale projects may exhibit greater ecological resilience.

To rigorously test the aforementioned hypotheses, our study will leverage the recently developed nationwide dataset of large-scale PV station distributions, with a spatial resolution of 10 m (Feng et al., 2024). In conjunction with this, we will employ the Normalized Difference Vegetation Index (NDVI) as a key metric for assessing vegetation greenness. By integrating these two RS datasets, we will conduct a comprehensive analysis of the NDVI across various regions and installation scales, thereby elucidating both the immediate disturbances and long-term ecological consequences of PV development. The primary objectives of our study are as follows:

1. To assess the ecological impacts and underlying mechanisms of PV installations in arid and humid regions.
2. To explore the impact of PV installation scale on ecosystem vitality and analyze optimal construction scales.

## 2 Materials and methods

### 2.1 Distribution data of PV station

The PV station dataset used in our study is the first publicly released 10 m resolution ground-based PV station grid dataset for China, which provides nationwide spatial distribution information on PV installations (Feng et al., 2024). Developed on the Google Earth Engine (GEE) platform, the dataset employs a random forest classifier and active learning strategies to achieve high-precision PV station distribution identification through the integration of multidimensional features. During the data construction process, ground-based samples covering the entire country were collected, and sample labeling was completed through field surveys and visual interpretation. Subsequently, spectral and texture features from Sentinel-2 RS imagery were extracted, alongside terrain-related characteristics such as slope and aspect, forming a multidimensional feature space for differentiation. Using the GEE platform, parallel predictions were conducted to generate the nationwide PV station distribution map. The final data validation indicated a classification accuracy exceeding 89%, demonstrating high technical reliability (Feng et al., 2024).

In comparison to previous datasets, this dataset offers substantial advantages: First, the 10 m spatial resolution provides a higher level of detail, making it particularly well-suited for nuanced investigations of PV installations across diverse climatic zones, including both arid and humid regions. Second, unlike datasets that rely on low resolution or insufficient sampling coverage, this dataset significantly enhances its reliability and utility through extensive ground-based samples and the integration of multidimensional feature modeling. Third, the rasterized format based on Sentinel-2 imagery supports temporal analysis and large-scale geographic assessments, providing a robust data foundation for evaluating the ecological impacts of USSE development. With its high-resolution spatial coverage and nationwide distribution map, this dataset is particularly well-positioned to facilitate comparative analyses of PV development in arid and humid regions.

**Figure 1:**
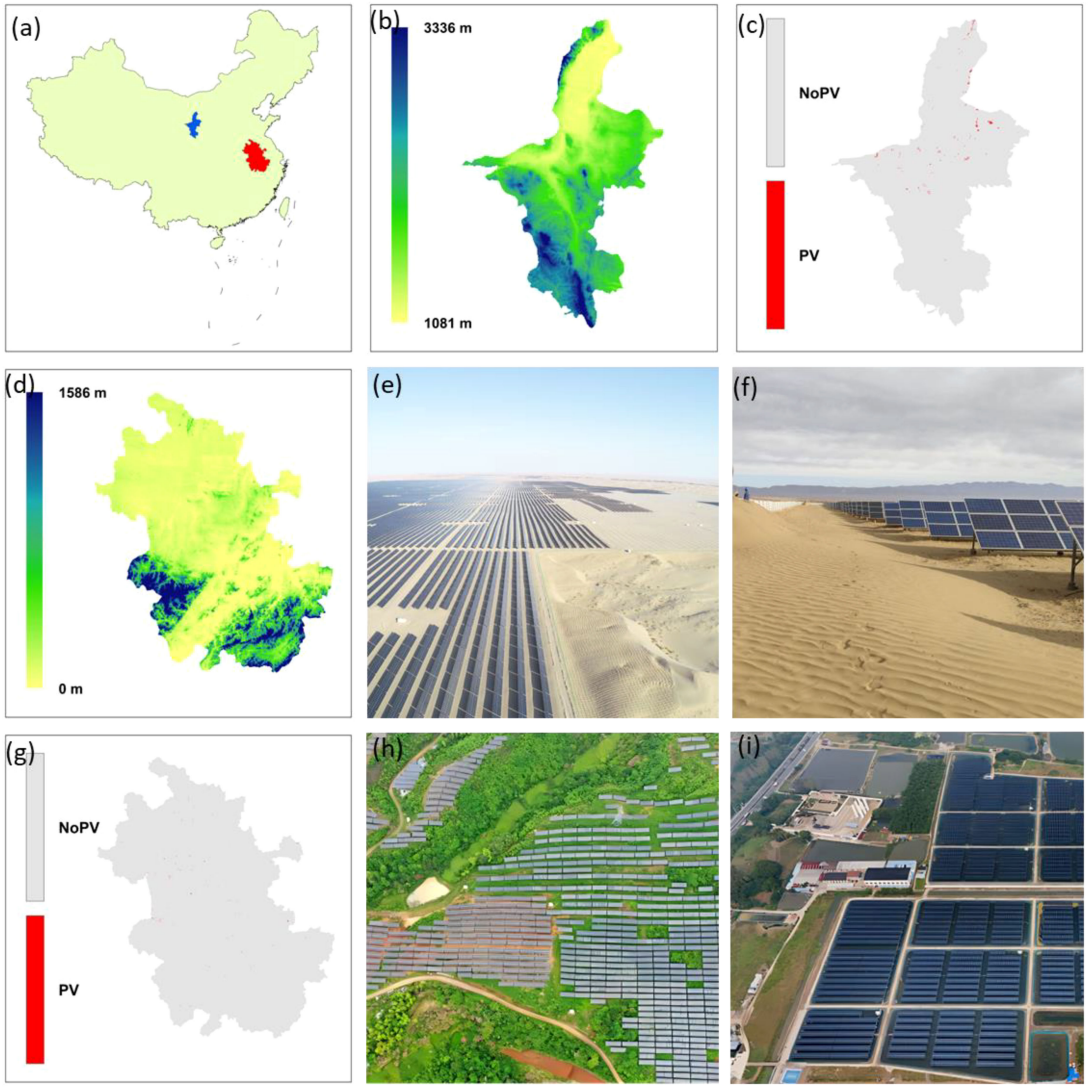
Geographic location, topography, and distribution of PV stations in typical arid (Ningxia) and humid (Anhui) regions. **(a)** Geographical locations of Ningxia (blue) and Anhui (red) within China; **(b)** Elevation distribution in Ningxia; **(c)** PV station distribution in Ningxia, with red areas indicating PV station locations. **(e, f)** Views of a PV station in the arid region. **(d)** Elevation distribution in Anhui; **(g)** PV station distribution in Anhui, with red areas indicating PV station locations. **(h, i)** Views of a PV station in the humid region.

The Ningxia and Anhui were selected as representative regions for investigating the ecological effects of USSE development in arid and humid regions, respectively. As shown in **Figure 1**, there are clear and significant differences between Ningxia and Anhui in terms of PV plant siting, topography, and land-use. These disparities provide an ideal experimental setting to study the ecological impacts of PV facilities under varying hydrothermal conditions. Ningxia, located in the northwestern part of China, is a typical arid region characterized by a landscape dominated by deserts and gobi, with low vegetation cover and relatively harsh natural conditions (**Figures 1b, c, e, f**). PV plants in this region are primarily located on desertified land and degraded grasslands (Zhang et al., 2021). In contrast, Anhui is markedly more humid, with land predominantly used for agriculture. The surrounding vegetation around PV installations is dense, representing high-biomass ecosystems (**Figures 1d, g–i**).

In this study, the dataset provided by Feng et al. (2024) was utilized to obtain high-resolution distribution data of PV stations in Ningxia and Anhui (**Figures 1c, g**), offering precise spatial information to explore the potential ecological risks and land-use conflicts associated with PV development in arid and humid regions.

### 2.2 Landsat NDVI dataset

Our study leverages the Landsat NDVI dataset, available through the GEE platform, spanning from 2019 to 2023, to comprehensively assess the ecological implications of PV facility deployment on vegetation greenness. The NDVI is a widely recognized and extensively utilized vegetation monitoring metric, offering distinct advantages in quantifying vegetation vitality and temporal dynamics (Yang et al., 2021). Its application is particularly well-suited for large-scale, long-duration studies, where it demonstrates exceptional efficacy in delivering reliable and robust insights into vegetation condition.

To ensure the comprehensiveness and accuracy of the analysis, we extracted the mean and maximum NDVI values for each time period. The NDVI_mean_ indicator reflects the overall state of vegetation greenness, and it is widely utilized globally, particularly in arid regions, where it is highly sensitive to vegetation monitoring (Yang et al., 2021). Meanwhile, NDVI_max_ represents the maximum photosynthetic potential of vegetation under optimal growth conditions, effectively mitigating potential disturbances such as cloud cover and providing an accurate representation of the vegetation’s peak growth status (Liu et al., 2023). By combining these two key indicators, our study enables a precise quantification of the disturbances to vegetation greenness caused by PV facilities, and provides insights into the regional differences between arid and humid regions. Specifically, the NDVI data from 2019 represents the baseline state prior to PV facility construction, while the data from 2020 captures the immediate post-construction changes. The data from 2021 to 2023 is used to assess the long-term environmental impacts. Moreover, the monthly mean (NDVI_mean_) values were also extracted to explore the seasonal variation in vegetation greenness resulting from PV facility construction.

Additionally, given that the Landsat data was originally at a 30 m resolution, whereas the photovoltaic data was at a 10 m resolution, we conducted a resampling of the NDVI data to a 10 m resolution to ensure spatial congruency

### 2.3 Statistical analysis

To accurately assess the disturbance effects of PV facilities on ecosystems, our study established a 500 m buffer zone extending beyond the grid cells of the PV installations, which was defined as the control area. The design of this buffer zone is based on the principle of spatial congruence, aiming to ensure a high degree of environmental similarity between the buffer zone and the PV development area, particularly in terms of soil and climatic conditions. In particular, when analyzing NDVI variations, the buffer zone serves as a baseline, facilitating a more precise comparison of the direct impact of PV facility construction on vegetation greenness.

To ensure the reliability and robustness of the results, our study focused exclusively on large-scale PV energy systems, systematically excluding PV installation grid cells with fewer than 100 pixels. This threshold was established by considering both the minimum effective area required for PV installations and the spatial distribution characteristics of the data, thereby optimizing the statistical power of the analysis while minimizing the potential for random noise introduced by small sample sizes. Furthermore, to explore the heterogeneous ecological impacts of PV installations at different scales, we classified the PV development areas into five categories based on pixel count: <2,500, 2,500–5,000, 5,000–7,500, 7,500–10,000, and >10,000. Subsequently, we computed the NDVI_mean_ and NDVI_max_ values for each category of PV installation areas and their corresponding buffer areas.

Moreover, we classified PV systems into two categories: >5000 pixels and <5000 pixels. Systems exceeding 5000 pixels represent large-scale installations with notable environmental impacts, while those under 5000 pixels are smaller systems with relatively limited ecological effects. Furthermore, the 5000-pixel threshold aligns with other classifications.

## 3 Results

**Figure 2:**
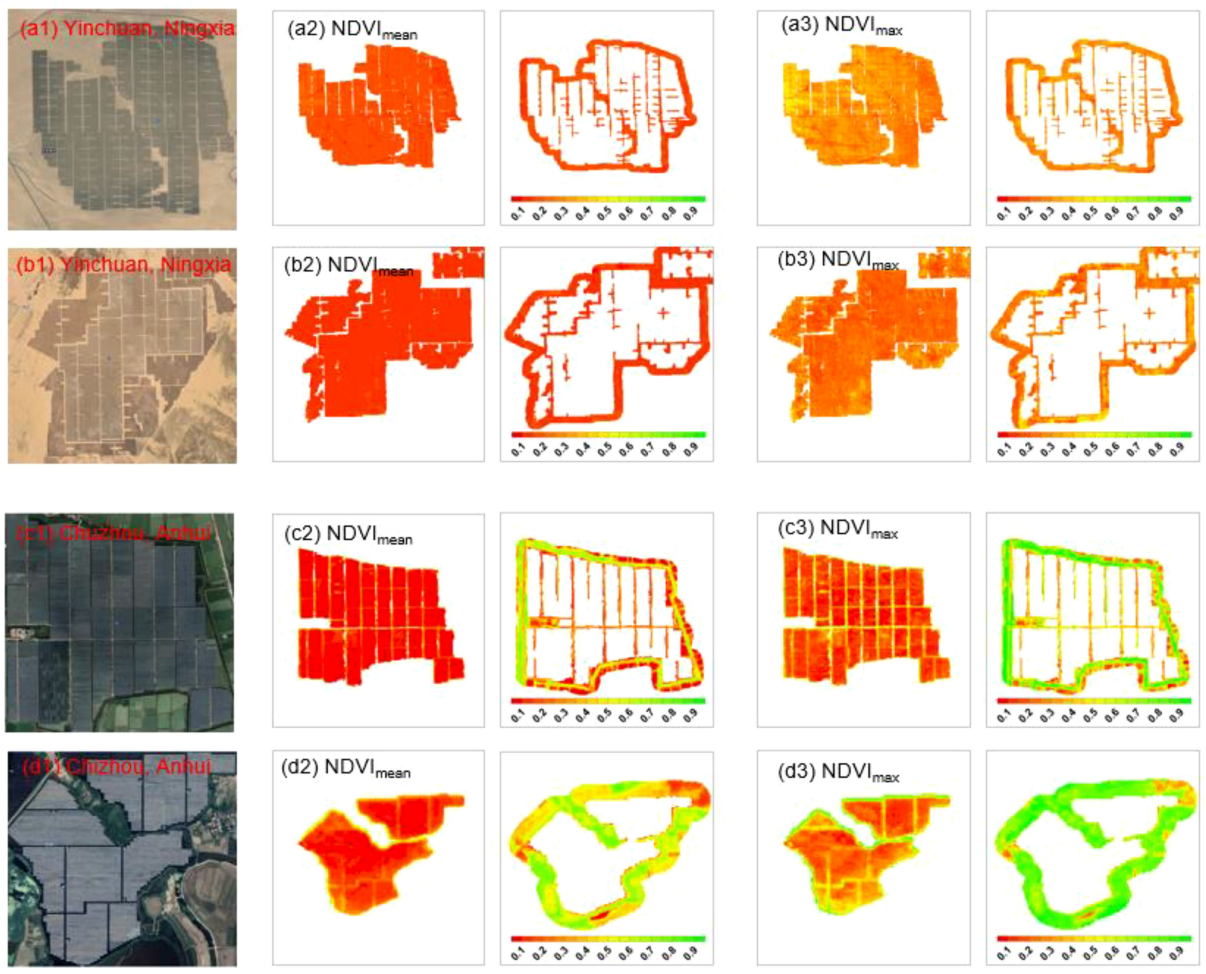
Spatial distribution of vegetation greenness for four USSEs in Ningxia **(a, b)** and Anhui **(c, d)**. The first column shows Google imagery of the PV stations, the second column displays NDVI_mean_ distribution within the PV development areas, the third column shows NDVI_mean_ distribution in the control areas, the fourth column presents NDVI_max_ distribution within the PV development areas, and the fifth column shows NDVI_max_ distribution in the control areas.

We randomly selected two sample PV stations, each covering more than 5,000 pixels, located in the arid region (Yinchuan, Ningxia; **Figures 2a, b**) and the humid region (Chizhou, Anhui; **Figures 2c, d**). The two PV stations in Ningxia are shown in **Figures 2a** and **2b**. The analysis revealed that the NDVI_mean_ within the PV development areas remained stable, ranging from 0.15 to 0.18. NDVI_max_ values were concentrated between 0.30 and 0.35. Compared to the PV development areas, the control areas exhibited slightly higher NDVI_mean_ and NDVI_max_, although the differences were not statistically significant.

The two PV stations in the humid region are shown in **Figures 2c** and **2d**. Both NDVI_mean_ and NDVI_max_ within the PV development areas are significantly lower than those in the control areas. Specifically, NDVI_mean_ dropped to below 0.2 following PV installation, and NDVI_max_ also decreased to less than 0.35. In contrast, NDVI_mean_ and NDVI_max_ in the two control areas were significantly higher than in the PV development areas, maintaining values above 0.4 and 0.6, respectively.

**Figure 3:**
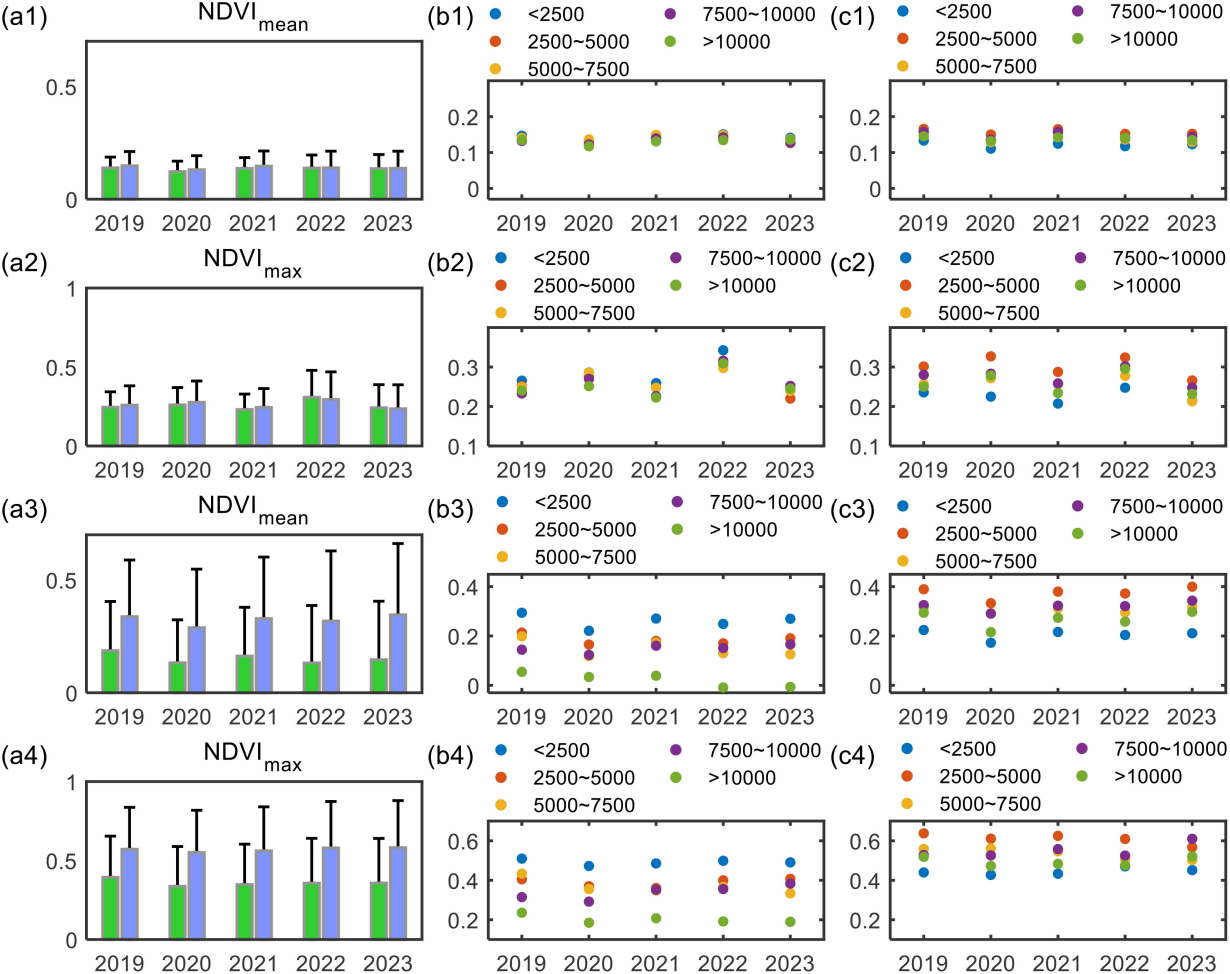
Annual dynamics and scale-dependent comparison of NDVI in PV development areas and their control areas. The left column shows the annual average NDVI_mean_ and maximum NDVI_max_ for the PV development areas (blue) and corresponding buffer areas (green), displaying changes in the arid region **(a1, a2)** and humid region **(a3, a4)**, with error bars representing the standard deviation of NDVI values. The middle column compares the NDVI across PV development areas with varying scales **(b1–b4)**. The right column compares the NDVI across buffer areas with varying scales **(c1–c4)**.

Subsequently, we observed starkly contrasting changes in ecosystem NDVI in different climatic zones following the construction of PV stations. In humid regions, the establishment of PV stations led to a dramatic decline in vegetation greenness, while in arid regions, the greenness remained relatively stable (**Figure 3a**).

Specifically, we found that the NDVI_mean_ values of the PV development area and the control area in Ningxia remained stable around 0.18 between 2019 and 2023, with annual variations of less than 0.05. The NDVI_max_ values exhibited a similar trend, consistently remaining around 0.35 each year. In contrast, after the construction of PV stations in Anhui, a marked and significant decline in NDVI was observed. For NDVI_mean_, the control area prior to PV development exhibited values ranging from 0.42 to 0.44, whereas post-construction values in the PV development area plummeted to 0.2, reflecting a dramatic decrease of up to 100%. NDVI_max_ shows similar trends. In the control areas of PV stations, NDVI_max_ typically exceeds 0.55. However, in the construction areas of PV stations, NDVI_max_ values decrease to below 0.4.

When comparing the differential impacts of PV station scales on vegetation greenness between PV development areas and control areas, as anticipated, we found that in the arid region, the impact of different PV station scales on NDVI_mean_ and NDVI_max_ was minimal. Across all five PV facility scales, NDVI_mean_ remained consistently around 0.15 for both the PV development and control areas in each year. NDVI_max_ exhibited greater variability across years, but no significant pattern emerged with respect to the scale of PV stations. In contrast, when comparing the NDVI dynamics of PV and control areas in the humid region, a clear pattern emerged: both NDVI_mean_ and NDVI_max_ decreased progressively as PV facility scale increased (**Figure 3b**). This pattern was not observed in the control areas (**Figure 3c**). Notably, in ultra-large-scale PV development areas (>10,000 pixels), the NDVI_mean_ approached 0, indicating complete vegetation loss and cessation of photosynthetic activity. In contrast, the NDVI_mean_ in control areas of the same scale consistently exceeded 0.2 across all years, highlighting a stark contrast with the PV development areas.

**Figure 4:**
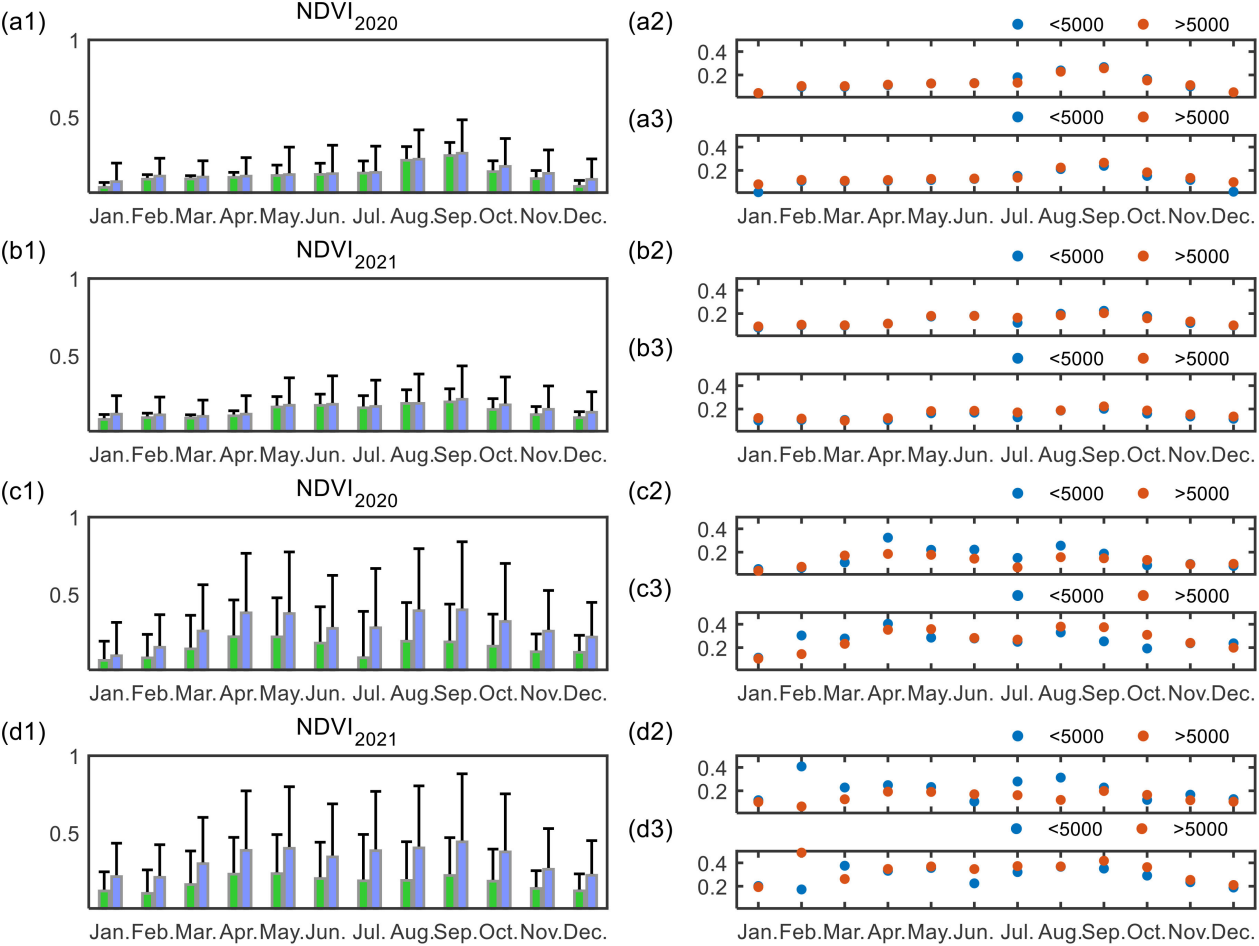
Monthly NDVI variations in PV development areas and control areas, with scale-based impact analysis. The left column displays the monthly average NDVImean for the PV development areas (blue) and buffer areas (green) in Ningxia **(a1, b1)** and Anhui **(c1, d1)** for 2020 and 2021. Panels **(a2, b2, c2)**, and **(d2)** show NDVI for different PV facility scales (<5000, >5000 pixels) in the facility zones. Panels **(a3, b3, c3)**, and **(d3)** show the corresponding NDVI distribution in buffer areas of varying scales.

Finally, we compared the seasonal differences in NDVI between 2020 and 2021, with 2020 being the year the data was provided and 2021 used as a reference year (**Figure 4**). It is evident that in the arid region, the NDVI in both the PV development area and the control area exhibited minimal seasonal variation, with differences generally not exceeding 0.05. In contrast, in the humid region, NDVI in the PV development area was consistently significantly lower than in the control area across all months, particularly between June and August.

Similarly, we compared the NDVI differences between PV stations with scales > 5,000 pixels and < 5,000 pixels. In the arid region, NDVI values corresponding to different PV station scales in both years were largely consistent across months, showing no significant differences. In contrast, the humid region exhibited a marked and significant variation, with NDVI differing between months following the construction of PV stations of different scales. Larger-scale PV installations resulted in significantly lower NDVI during the growing season (April to October) compared to smaller-scale installations, a pattern not observed in the control areas. In summary, the impact of PV facility scale was more pronounced in the humid region, with comparisons of buffer zone NDVI further highlighting regional differences in ecosystem responses to PV development.

## 4 Discussion

### 4.1 Low disturbance of photovoltaic development on vegetation greenness in arid regions

The deployment of USSE systems has increasingly been recognized as a pivotal strategy in mitigating climate change, leading to heightened scrutiny of its environmental consequences (Armstrong et al., 2016; Barron-Gafford et al., 2016). By examining interannual, scale-specific, and seasonal variations in vegetation indices, specifically NDVI_mean_ and NDVI_max_, derived from RS data, we revealed significant ecological disparities in the effects of USSE development across diverse climatic zones. Consistent with our hypothesis, our findings indicate a pronounced decline in vegetation greenness within PV development areas in humid regions, whereas in arid regions, vegetation greenness exhibits relative stability.

In the arid region, the construction of PV power stations has led to remarkable stability in both NDVI_mean_ and NDVI_max_, reflecting the ecosystem’s strong adaptability to solar energy development. This stability may be closely linked to the physiological resilience of vegetation systems in arid regions, as well as the inherently low background greenness (Li et al., 2024; Zhang et al., 2024b). In Ningxia, the NDVI_mean_ value remained stable at approximately 0.18 both before and after PV installation, with annual variations of less than 0.05. The observed stability in arid regions may stem from the already low vegetation coverage, which limits the direct impact of PV development on plant growth. Additionally, the shading effect of PV panels likely mitigates surface temperature changes and reduces water evaporation to some extent (Yue et al., 2021), thus alleviating vegetation stress. Notably, whether for PV stations with more than 5,000 pixels or smaller-scale installations, the NDVI_mean_ and NDVI_max_ in both the construction and control areas in the arid region exhibited no significant differences. This suggests that PV infrastructure in areas with extremely low vegetation cover, such as deserts and Gobi regions, may result in minimal ecological disruption. The shading effect in arid regions has a very negligible impact on vegetation, as the PV construction’s shading effect does not alter the existing low NDVI and low albedo conditions in these areas.

Vegetation in arid regions typically exhibits low coverage and is adapted to extreme environmental conditions, making it highly resilient to anthropogenic disturbances (Xia et al., 2023). Studies conducted in arid regions have shown that the widespread presence of biological soil crusts and drought-tolerant shrubs effectively stabilizes the soil and reduces water evaporation (Potter and Weigand, 2016; Luo et al., 2023). Even with the land disturbances caused by PV facility construction, the ecological functions of vegetation in these regions remain highly adaptable (Luo et al., 2023). Furthermore, NDVI is significantly correlated with chlorophyll fluorescence levels, and the installation of PV panels may indirectly maintain vegetation’s photosynthetic efficiency by locally inhibiting surface water evaporation (Xia et al., 2022). Additionally, the impact of soil disturbance is limited due to the pre-existing low vegetation cover in these areas, ensuring minimal changes in NDVI.

The shading effect of PV panels on the microclimate has both positive and negative ecological impacts (Lambert et al., 2023). On the one hand, the shading effect reduces direct sunlight exposure, lowers surface temperatures, and decreases evaporation rates, thereby alleviating water stress to some extent (Yue et al., 2021). For instance, Liu et al. (2020) demonstrated that temperatures beneath PV panels during the growing season were 40% lower than those above the panels, with a significant reduction in light radiation. This may be attributed to the shading effect improving surface microhabitats, which in turn provides a positive stimulus for the growth of drought-tolerant plants (Liu et al., 2020). However, shading can also reduce light intensity, potentially inhibiting the growth of plants that depend on high-light levels. In arid regions, however, the vegetation’s strong adaptability to light intensity typically mitigates this negative effect (Yang et al., 2023a), with the positive effects generally outweighing the drawbacks. Overall, the contribution of the shading effect of PV panels to NDVI_mean_ and NDVI_max_ is primarily reflected in its role in stabilizing the microclimate, rather than in significantly enhancing vegetation greenness.

The impact of PV installations of varying scales on NDVI_mean_ and NDVI_max_ in arid regions is negligible. Research on PV stations in China’s arid regions reveals that even large-scale PV installations do not significantly affect vegetation cover, reflecting the remarkable resilience of arid ecosystems to large-scale land use changes. Despite the direct effects of PV facilities on soil and vegetation distribution (Zhang et al., 2024b), there is no discernible reduction in vegetation coverage or greenness in these areas. This phenomenon suggests that the characteristics of low-coverage vegetation, combined with the disturbance resistance of arid-region flora, render the scale of PV facilities relatively insensitive to regional ecological impacts. While large-scale PV installations may induce more pronounced changes in the microclimate, their negative effects on vegetation greenness remain largely imperceptible in arid regions (Wang et al., 2024b).

Moreover, although the construction of USSE systems induces localized surface disturbances, ecosystems in arid regions exhibit a high degree of resilience (Zhang et al., 2024a). Studies in arid regions have confirmed that vegetation in these areas can recover to pre-construction levels within 5 to 6 years, likely due to the natural adaptability and rapid recovery capabilities of the flora (Ma et al., 2023). Furthermore, the regeneration of biological soil crusts and the expansion of drought-tolerant plant root systems contribute significantly to the ecological recovery process (Luo et al., 2023).

### 4.2 Ecological vulnerability in photovoltaic development in humid regions

In contrast to arid regions, PV development in humid regions has led to a marked and rapid decline in vegetation greenness and productivity. Taking Anhui as an example, the NDVI_mean_ dropped precipitously from 0.42–0.44 to below 0.2 following PV construction, indicating a significant reduction in vegetation cover. The high biomass characteristics of vegetation in humid regions render it particularly sensitive to environmental disturbances: abundant water resources and soil nutrients support dense vegetation, yet the construction of PV facilities directly removes vegetation through land disturbance (Grippo et al., 2015; Sánchez-Zapata et al., 2016). PV development alters rainfall distribution, increases surface runoff (Pisinaras et al., 2014), and reduces groundwater recharge, ultimately diminishing the availability of water for vegetation. In this context, while the shading effect of PV panels alleviates high temperatures to some extent, it further suppresses the high-productivity plants in humid regions that rely heavily on photosynthesis (Choi et al., 2021). These complex interactions culminate in the sharp decline of vegetation greenness and exacerbate the vulnerability of ecosystems during the growing season in humid regions.

The scale effect of PV installations in humid regions exacerbates this issue. We found that in areas covered by ultra-large-scale PV facilities (>10,000 pixels), the NDVI_mean_ nearly dropped to zero, indicating a complete loss of ecosystem photosynthetic activity and a near-total disappearance of vegetation. In contrast, smaller-scale installations had relatively lighter ecological impacts, yet they still significantly altered local ecological balances. This finding not only confirms the sensitivity of vegetation systems in humid regions to scale-related disturbances, but also underscores the importance of controlling the size of PV installations to protect ecosystems in these areas. Moreover, the prolonged presence of PV facilities further extends the ecosystem recovery period, causing long-term damage to species diversity and ecosystem services (Grippo et al., 2015; Yang et al., 2023b). Plant communities in humid regions often rely on stable environmental conditions, and their recovery capacity is much lower than that of arid zone ecosystems. These long-term ecological consequences warrant further attention and in-depth investigation.

In summary, the comparison between PV development in arid and humid regions offers critical insights into the adaptive capacities of different ecosystems. Compared to arid regions, vegetation systems in humid regions exhibit greater vulnerability and lower resilience. In arid regions, due to the low cover of vegetation and adaptive ecological characteristics, changes in NDVI_mean_ and NDVI_max_ remain within the natural variability, even under the coverage of large-scale PV facilities. However, the high density of vegetation and its dependency on water resources in humid regions make it more susceptible to disturbances caused by PV installations (Yang et al., 2023b). This disparity not only highlights the high ecological costs in humid regions but also underscores the potential ecological advantages of PV development in arid regions with respect to sustainability (Zhang et al., 2024a). Future research should further explore the optimization of PV facility design in humid regions, such as enhancing light permeability and controlling scale, to mitigate its negative impacts on ecosystems.

### 4.3 Opportunities for photovoltaic development in arid regions and ecological protection strategies in humid regions

Land-use conflicts and the trade-offs between ecological services are critical considerations in the expansion of PV infrastructure, particularly in regions with starkly different ecological service capacities and land-use pressures, such as arid and humid regions (Dias et al., 2019; Zhang et al., 2023; Song et al., 2024). Arid regions, characterized by land degradation and the availability of vast unutilized wastelands, present substantial strategic opportunities for the deployment of PV facilities (Zhang et al., 2024a). In recent years, the Chinese government has actively facilitated the development of PV projects in these areas through targeted policies. For instance, the “List of Key Large-Scale Wind and Photovoltaic Base Projects, Focusing on Desert, Gobi, and Desertified Regions” released in 2021 (https://www.ndrc.gov.cn), emphasizes the use of degraded land resources in provinces such as Ningxia, Gansu, and Qinghai, aiming to promote energy transition while simultaneously achieving ecological conservation objectives.

In arid regions, where land utilization value is relatively low, the intrinsic ecological services are limited, and vegetation coverage is sparse, leading to a diminished sensitivity to environmental disturbances. In such regions, the shading effect of PV installations significantly ameliorates the local microclimate, reducing soil moisture evaporation and alleviating the risks of aeolian erosion (Hernandez et al., 2014; Liu et al., 2020; Wang and Gao, 2023). Moreover, when integrated with ecological restoration strategies such as vegetative regeneration and the rehabilitation of biological soil crusts, PV development in arid regions not only enhances carbon sequestration potential but also markedly improves soil water retention and bolsters broader ecosystem service functions (Luo et al., 2023; Wang et al., 2024a).

In sharp contrast to the developmental opportunities afforded by low-competition lands in arid regions, the land resources in humid regions are subject to significant development conflicts due to their high-value utilization in agriculture, forestry, and wetlands (Song et al., 2024). The dense biomass of vegetation and the complex array of ecosystem services—including food production, hydrological regulation, and carbon sequestration—make the ecological costs of PV development in these regions substantially higher (Choi et al., 2021). The establishment of PV infrastructure may encroach on valuable agricultural land or forested areas, exacerbating soil erosion, diminishing groundwater recharge, and thereby disrupting regional hydrological cycles and balance (Zhang et al., 2023). Wetlands, vital ecosystems themselves, may also suffer from the loss of biodiversity maintenance and climate regulation functions due to the disturbances caused by PV installations (Grippo et al., 2015). Moreover, the construction of PV facilities in humid regions typically involves vegetation removal, a process that releases stored carbon in soils and biomass, thereby amplifying the region’s greenhouse gas emissions.

Thus, PV development in China must achieve a nuanced equilibrium between ecological conservation and energy transition (Zhang et al., 2023; Li et al., 2024). Our findings suggest that future PV expansion should center on arid regions, while humid regions should adopt a policy of constrained development. The expansive deserts, gobi, and barren-lands of arid regions, characterized by low land-use competition and high ecological resilience, present optimal conditions for large-scale PV deployment. These regions offer an opportunity to leverage degraded land, integrating shading effects and vegetation regeneration technologies to create an “ecological photovoltaic” framework, facilitating the concurrent advancement of ecological restoration and energy production. Furthermore, policy initiatives should continue to reinforce support for arid regions such as Ningxia, Qinghai, and Gansu, promoting the development of large-scale PV installations and the optimization of transmission infrastructure to enhance the efficient delivery of clean energy. In contrast, humid regions, with their high-value ecological services—including agriculture, forestry, and wetlands—are particularly vulnerable to large-scale PV expansion, which could compromise vital ecosystem functions such as food production, hydrological regulation, and carbon sequestration (Hernandez et al., 2014; Zhang et al., 2023). By adopting this spatially tailored development strategy, China can achieve a dynamic balance between energy production in arid regions and ecological preservation in humid regions, setting a global benchmark for sustainable energy development.

## 5 Conclusion

Our study provides a comprehensive analysis of the construction of USSE projects in arid (Ningxia) regions and humid (Anhui) regions and their differential impacts on ecosystem vegetation condition, highlighting significant variations in vegetation greenness (NDVI_mean_ and NDVI_max_) driven by regional ecological characteristics and the scale of PV development. Our findings demonstrate that PV development in arid regions exerts minimal disturbance on vegetation greenness, with NDVI values remaining exceptionally stable before and after the installation of PV facilities, showing interannual variations of less than 0.05. In stark contrast, in humid regions, the deployment of PV infrastructure induces pronounced ecological degradation, with both NDVI_mean_ and NDVI_max_ declining substantially, by as much as 50%. During the growing season (April–October), the suppressive effects of large-scale PV facilities on vegetation greenness are particularly evident, with some areas experiencing complete cessation of photosynthetic activity. Moreover, the scale of PV facilities exhibits region-specific ecological responses: while the expansion of PV development in arid regions has negligible effects on vegetation condition. In humid regions, increasing facility size corresponds to a marked decline in NDVI, underscoring the heightened vulnerability of humid ecosystems to large-scale interventions. Therefore, regulating the scale of PV facilities is critical for ecological conservation in humid regions.

In summary, our study confirms that the ecological impacts of PV installations exhibit significant variability depending on hydrothermal conditions. The development of USSE in arid regions demonstrates higher ecological adaptability and sustainability, whereas humid regions face considerably greater ecological costs. Therefore, future PV development should be prioritized in arid regions, where the ecological carrying capacity is more robust. Additionally, careful optimization of PV design and management strategies is crucial to minimize the disruption of high-value ecosystems in humid regions.

## Data Availability

The original contributions presented in the study are included in the article/supplementary material. Further inquiries can be directed to the corresponding author.
